# Delving into LLM-assisted writing in biomedical publications through excess vocabulary

**DOI:** 10.1126/sciadv.adt3813

**Published:** 2025-07-02

**Authors:** Dmitry Kobak, Rita González-Márquez, Emőke-Ágnes Horvát, Jan Lause

**Affiliations:** ^1^ Hertie Institute for AI in Brain Health, University of Tübingen, 72076 Tübingen, Germany.; ^2^ Northwestern University, Evanston, 60208 IL, USA.

## Abstract

Large language models (LLMs) like ChatGPT can generate and revise text with human-level performance. These models come with clear limitations, can produce inaccurate information, and reinforce existing biases. Yet, many scientists use them for their scholarly writing. But how widespread is such LLM usage in the academic literature? To answer this question for the field of biomedical research, we present an unbiased, large-scale approach: We study vocabulary changes in more than 15 million biomedical abstracts from 2010 to 2024 indexed by PubMed and show how the appearance of LLMs led to an abrupt increase in the frequency of certain style words. This excess word analysis suggests that at least 13.5% of 2024 abstracts were processed with LLMs. This lower bound differed across disciplines, countries, and journals, reaching 40% for some subcorpora. We show that LLMs have had an unprecedented impact on scientific writing in biomedical research, surpassing the effect of major world events such as the COVID pandemic.

## Teaser

Excess words track LLM usage in biomedical publications.

## INTRODUCTION

When the world changes, human-written text changes. Major events like wars and revolutions affect word frequency distributions in text corpora (*1*). The rise and fall of scientific disciplines are traceable in scholarly writing (*2*, *3*). Do technological advances leave a similar footprint on our writing?

With the release of ChatGPT in November 2022, human writing underwent an unprecedented change: For the first time, a large language model (LLM) was widely available that could generate and revise texts with human-like performance in several domains—including academia (*4*), where many have hoped that LLMs might lead to more equity (*5*). Many researchers have since integrated LLMs in their daily writing tasks (*6*) and even coauthored papers with LLMs (*7*). This has led to worries about research integrity, factual mistakes in LLM-generated content (*8*–*12*), and misuse of LLMs by so-called paper mills that produce fake publications (*13*). These worries sparked attempts to track the footprint of LLM-assisted writing in scientific texts.

Recent approaches attempting to quantify the increasing use of LLMs in scientific papers all build on the idea that LLM-written text differs from human-written text (*14*, *15*) and fall in three groups. One group of studies used LLM detectors (*16*), which are blackbox models trained to detect LLM writing based on ground-truth human and LLM texts (*17*–*20*). Another group of works explicitly modeled word frequency distribution in scientific corpora as a mixture distribution of texts produced by humans and LLMs, again estimated using ground-truth human and LLM texts (*21*–*24*). The third group of studies relied on lists of marker words, known to be overused by LLMs, which are typically stylistic words unrelated to the text content (*19*, *25*, *26*).

All of these approaches share one common limitation: They require a ground-truth training set of LLM- and human-written texts. Usually, human-written texts are obtained from pre-LLM years, while LLM-written texts are generated by a set of prompts. This setup can introduce biases (*16*), as it requires assumptions on which models scientists use for their LLM-assisted writing, and how exactly they prompt them. Furthermore, work based on LLM detector models suffers from their blackbox nature, as it does not allow further interrogation of the results on the word level, which can make their interpretation difficult. All existing work focuses on detecting LLM texts, and none has attempted to systematically compare or relate LLM-induced changes in scientific writing to previous shifts in scholarly texts. This begs the question whether the nature and magnitude of the observed changes are comparable to changes that regularly occur due to changing fashions, rising research topics, and global events such as the COVID-19 pandemic—or if LLMs affect scientific writing in an unprecedented way.

Here, we suggest a data-driven approach to track LLM usage without requiring a labeled corpus of human-written versus model-generated texts. We were inspired by studies of excess mortality (*27*–*29*) that looked at the excess of fatalities during the COVID pandemic compared to pre-COVID mortality. We adapt this idea to LLM-induced changes in word usage and track the excess use of words after the release of ChatGPT-like LLMs compared to pre-LLM years. Applying this analysis to the corpus of more than 15 million 2010–2024 biomedical abstracts from the PubMed library allowed us to track changes in scientific writing over the past decade in the large field of biomedical research. We found that the LLM-induced changes were unprecedented in both quality and quantity.

## RESULTS

### Excess words indicate widespread LLM usage

We downloaded all PubMed abstracts until the end of 2024 and used all 15.1 million English-language abstracts from 2010 onward (after cleaning them from contaminating strings, see Materials and Methods). We then computed the matrix of word occurrences that shows which abstracts contain which words, resulting in a 15,103,888 × 273,112 sparse binary matrix. For each word and each year, we found the number of abstracts in that year that the word appeared in and obtained its occurrence frequency *p* by normalizing with the total number of papers published in that year. Our main analysis focused on 26,657 words with *p* > 10^−4^ in both 2023 and 2024. With more than 1 million abstracts per year, this corresponds to >100 usages per year.

**Fig. 1.**
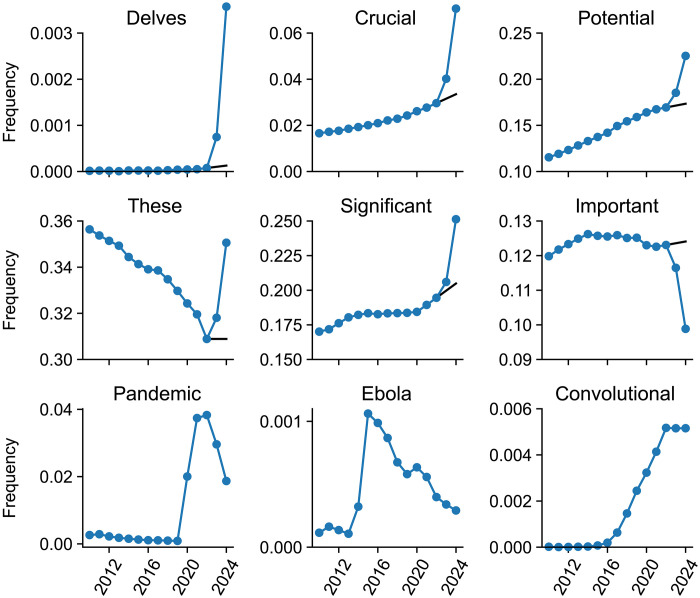
Frequencies of PubMed abstracts containing several example words. Black lines show counterfactual extrapolations from 2021–2022 to 2023–2024. We manually selected words to illustrate the diversity of frequency time courses. The first six words are affected by LLMs; the last three relate to major events that influenced scientific writing and are shown for comparison.

Some words strongly increased their occurrence frequency in 2023–2024 (Fig. 1). To quantify this increase, we calculated counterfactual expected frequency in 2024 based on the linear extrapolation of word frequencies in 2021 and 2022 (see Materials and Methods). Note that we did not use 2023 frequencies for calculating the expected frequency, because they could already have been affected by LLM usage. Comparing the empirical 2024 frequency *p* with the expected 2024 frequency *q*, we obtained the excess frequency gap δ = *p* − *q* and the excess frequency ratio *r* = *p*/*q* as two measures of excess usage. These two measures are complementary. The frequency gap is well-suited to highlight excess usage of frequent words, while the frequency ratio points to the excess usage of infrequent words. For example, frequency increases from 0.001 to 0.01 and from 0.5 to 0.6 are both noteworthy. Yet, the former frequency increase is captured by a high *r* value, whereas the latter has a high δ value.

**Fig. 2.**
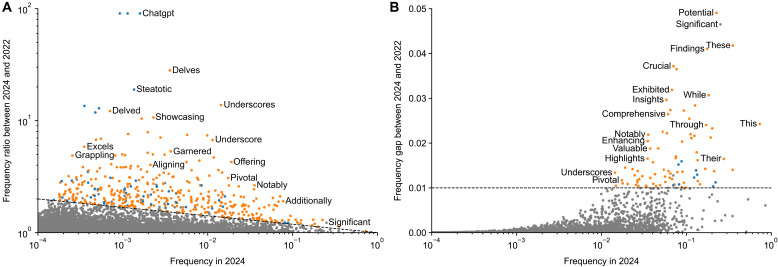
Words showing increased frequency in 2024. (**A**) Frequencies in 2024 and frequency ratios (*r*). Both axes are on a log scale. Only a subset of points are labeled for visual clarity. The dashed line shows the threshold defining excess words (see text). Words with *r* > 90 are shown at *r* = 90. Excess words were manually annotated into content words (blue) and style words (orange). (**B**) The same but with frequency gap (δ) as the vertical axis. Words with δ > 0.05 are shown at δ = 0.05.

Across all 26,657 words, we found many with strong excess usage in 2024 (Fig. 2). Less common words with strong excess usage included delves (*r* = 28.0), underscores (*r* = 13.8), and showcasing (*r* = 10.7), together with their grammatical inflections (Fig. 2A). More common words with strong excess usage included potential (δ = 0.052), findings (δ = 0.041), and crucial (δ = 0.037) (Fig. 2B).

Is this unusual, or do similar frequency changes happen every year? For comparison, we did the same analysis for all years from 2013 to 2023 (figs. S1 to S4). We found words like ebola with *r* = 9.9 in 2015 and zika with *r* = 40.4 in 2017, but from 2013 until 2019, no single word has ever shown excess frequency gap δ > 0.01. This changed during the COVID pandemic: In 2020–2022, words like coronavirus, covid, lockdown, and pandemic showed very large excess usages (up to *r* > 1000 and δ = 0.06), in agreement with the observation that the COVID pandemic had an unprecedented effect on biomedical publishing (*30*).

**Fig. 3.**
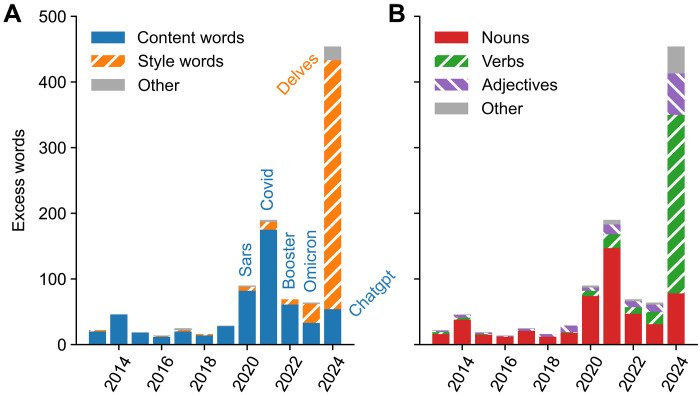
Number of excess words per year. (**A**) Decomposed into the excess content words and excess style words. In each year, we show, as an example, the word with the highest frequency ratio *r* among excess words with *P* > 0.0015 and *r* > 3 (in blue). (**B**) Decomposed into nouns, verbs, and adjectives. This figure is designed after *The Economist*’s coverage of our preprint (https://economist.com/science-and-technology/2024/06/26/at-least-10-of-research-may-already-be-co-authored-by-ai).

To compare the size of excess vocabulary between years, we defined as excess words all words with δ > 0.01 or ${\text{log}}_{10}r>\frac{{\text{log}}_{10}2}{4}{\text{log}}_{10}p$ where *p* is the frequency in 2024 (see dashed lines in Fig. 2); these thresholds were chosen such that most words in pre-COVID years were well below (figs. S1 to S4). The number of excess words showed a marked rise during the COVID pandemic (up to 190 words in 2021) followed by an even larger rise (to 454) in 2024 (Fig. 3), roughly 1 year after ChatGPT was released. Note that this counts grammatical inflections (such as delve, delves, delving, and delved or mask and masks) multiple times. Counting only unique word lemmas still put 2024 on a clear first place: 343 unique lemmas in 2024 versus 180 in 2021 (fig. S5).

We manually annotated all 900 unique excess words from 2013 to 2024 into content words (51.3%), like masks or convolutional, and style words (45.2%), like intricate or notably (and a small number of ambiguous words; see Materials and Methods). The excess vocabulary during the COVID pandemic consisted almost entirely of content words (such as respiratory, remdesivir, etc.), whereas the excess vocabulary in 2024 consisted almost entirely of style words (Fig. 3A). We also manually assigned part of speech to each excess word. Content words were predominantly nouns (79.2%), and hence most excess words before 2024 were nouns. In contrast, of all 379 excess style words in 2024, 66% were verbs, and 14% were adjectives (Fig. 3B).

### Combining excess words puts a lower bound on LLM usage

The unprecedented increase in excess style words in 2024 allows to use them as markers of LLM usage. Each frequency gap δ for a single marker word gives a lower bound on the fraction of abstracts that went through LLMs in 2024. For example, δ = 0.052 for the LLM style marker word potential means that, in 2024, there were 5.2 percentage points more abstracts containing that word than expected based on the 2021–2022 data, suggesting that at least 5.2% of all abstracts in 2024 went through an LLM. We reasoned that combining multiple marker words together can increase the lower bound.

For a given set of marker words *G*, we computed its frequency gap $\Delta_{G}=P_{G}-Q_{G}$ where $P_{G}$ and $Q_{G}$ are the observed and the expected fractions of abstracts containing at least one of the words from *G*. As above, we interpret $\Delta_{G}$ as a lower bound on the LLM usage. Note that this does not assume independence between words in *G*. To avoid a computationally expensive combinatorial search for a set of words with the highest $\Delta_{G}$ , we used two independent heuristics to form two separate sets, focusing on either rare or common style excess words.

**Fig. 4.**
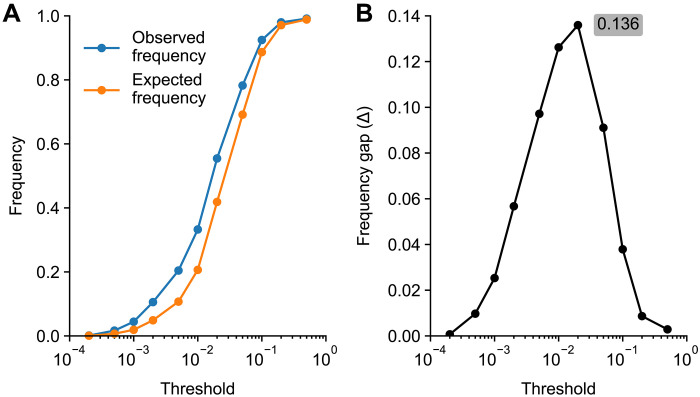
Combining excess style words yields a larger frequency gap. (**A**) Observed frequency (*P*) and counterfactual expected frequency (*Q*) in 2024 of abstracts containing at least one of the excess style words from 2024 with frequency *p* below a given threshold. (**B**) The frequency gap Δ = *P* − *Q* as a function of the threshold.

**Fig. 5.**
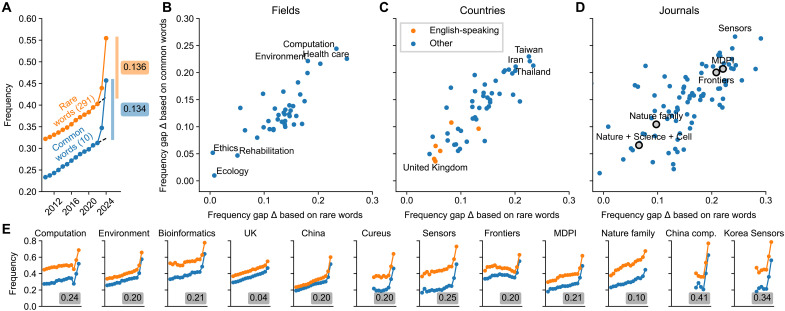
Frequency gaps estimated for various subcorpora. (**A**) Frequency of abstracts containing at least one word from a given word group. (**B**) Frequency gap estimates for various fields identified based on journal names (*30*). (**C**) Frequency gap estimates for various countries. (**D**) Frequency gap estimates for various journals. Gray circles show multiple journals grouped together. (**E**) Frequencies as in (A) for various PubMed subsets; $\Delta =\left(\Delta_{\text{common}}+\Delta_{\text{rare}}\right)/2$ values are shown.

To create the rare set, we grouped all 2024 excess style words with frequency *p* < *T* and computed the frequency gap $\Delta_{\text{rare}}$ as a function of threshold *T* (Fig. 4). We obtained the highest $\Delta_{\text{rare}}$ value with *T* = 0.02, resulting in a set of 291 words (fig. S6). The frequency gap was $\Delta_{\text{rare}}=0.136$ , putting the lower bound on the LLM usage in 2024 at 13.6% (Fig. 5A). This is only a lower bound because some of the abstracts that did go through an LLM may fail to contain any of the style words we selected here (see Discussion).

To create the common set, we grouped together excess style words with high individual δ values, manually adjusting the selection to maximize the frequency gap $\Delta_{\text{common}}$ . This led to the following set of 10 words: across, additionally, comprehensive, crucial, enhancing, exhibited, insights, notably, particularly, and within. This set yielded a very similar frequency gap as the rare words: $\Delta_{\text{common}}=0.134$ (Fig. 5A).

Both sets of words have their advantages. The advantage of the rare set is that it was produced automatically without any manual search and is hence free of researcher bias. The advantage of the common set is that it consists of only a few words, increasing the transparency of our procedure. As the rare and the common sets were nonoverlapping, both serve as an independent estimate of the lower bound. The fact that we arrived at very similar estimates with these two approaches underscores our results’ robustness. Averaging the two estimates, we obtained $\Delta =\left(\Delta_{\text{common}}+\Delta_{\text{rare}}\right)/2=0.135$ as our final estimate of the lower bound of LLM usage (13.5%).

For comparison, using the set of four excess content words from 2021, covid, pandemic, coronavirus, and sars (any scientific paper on COVID-19 likely contained at least one of these four words in its abstract), yielded frequency gap Δ = 0.069. This shows that the LLM usage in 2024 was at least two times higher than the size of COVID-related literature at its peak in 2021.

### Lower bounds differed between subcorpora

We performed the same analysis as above by various subgroups of PubMed papers. We computed frequency gaps $\Delta_{\text{common}}$ and $\Delta_{\text{rare}}$ for different biomedical fields, affiliation countries, journals, and men and women among the first and the last authors, inferred from their first names (see Materials and Methods). Note that we based all our Δ estimates on the same two sets of excess style words as before. While there may be additional subcorpus-specific excess words, using the same sets of marker words ensures a fair and consistent comparison between the subcorpora.

We found pronounced heterogeneity among most of these categories. Computational fields like computation and bioinformatics showed Δ ≈ 0.20 (Fig. 5B). Among countries, some English-speaking countries like United Kingdom and Australia showed Δ ≈ 0.05, while countries like China, South Korea, and Taiwan showed Δ ≈ 0.20 (Fig. 5B). The difference between inferred genders was minor (0.11 for male and 0.10 for female, both for the first and the last authors).

Among individual journals, we found very high Δ values, e.g., 0.25 for *Sensors* [an open access journal published by Multidisciplinary Digital Publishing Institute (MDPI)] and 0.20 for *Cureus* (an open access journal with simplified review process, published by Springer Nature). We analyzed several groups of journals pooled together and found high Δ for MDPI (0.21) and Frontiers (0.20) journals. For very selective high-prestige journals like *Nature*, *Science*, and *Cell* and for Nature family journals, Δ was much lower (0.07 and 0.10, respectively), suggesting that easily detectable LLM usage was negatively correlated with perceived prestige.

To find subgroups with the strongest effect, we looked at intersections of different groups and found Δ = 0.34 for papers from South Korea published in *Sensors* and Δ = 0.41 for computation papers from China. An even more fine-grained analysis based on *t*-distributed stochastic neighbor embedding (*t*-SNE) (*31*) of 2022–2024 papers showed some areas with local Δ ≈ 0.50 (fig. S7), such as, for example, a cluster of papers on deep learning–based object detection, with predominantly Chinese affiliations and with the majority published in MDPI’s *Sensors*.

## DISCUSSION

### Summary

Here, we leveraged excess word usage to show how LLMs have affected scientific writing in biomedical research. We found that the effect was unprecedented in quality and quantity: Hundreds of words have abruptly increased their frequency after ChatGPT-like LLMs became available. In contrast to previous shifts in word popularity, the 2023–2024 excess words were not content-related nouns but rather style-affecting verbs and adjectives that LLMs prefer.

Our analysis is performed on the corpus level and cannot identify individual abstracts that may have been processed by an LLM. Still, the following examples from three real 2023 abstracts illustrate the LLM-style flowery language:

1) By meticulously delving into the intricate web connecting […] and […], this comprehensive chapter takes a deep dive into their involvement as significant risk factors for […].

2) A comprehensive grasp of the intricate interplay between […] and […] is pivotal for effective therapeutic strategies.

3) Initially, we delve into the intricacies of […], accentuating its indispensability in cellular physiology, the enzymatic labyrinth governing its flux, and the pivotal […] mechanisms.

Our analysis of the excess frequency of such LLM-preferred style words suggests that at least 13.5% of 2024 PubMed abstracts were processed with LLMs. With ~1.5 million papers being currently indexed in PubMed per year, this means that LLMs assist in writing at least 200,000 papers per year. This estimate is based on LLM marker words that showed large excess usage in 2024, which strongly suggests that these words are preferred by LLMs like ChatGPT that became popular by that time. This is only a lower bound: Abstracts not using any of the LLM marker words are not contributing to our estimates, so the true fraction of LLM-processed abstracts is likely higher.

### Interpretation and limitations

Our estimated lower bound on LLM usage ranged from below 5% to more than 40% across different PubMed-indexed research fields, affiliation countries, and journals. This heterogeneity could correspond to actual differences in LLM adoption. For example, the high lower bound on LLM usage in computational fields (20%) could be due to computer science researchers being more familiar with and willing to adopt LLM technology. In non-English speaking countries, LLMs can help authors with editing English texts, which could justify their extensive use. Last, authors publishing in journals with expedited and/or simplified review processes might be grabbing for LLMs to write low-effort articles.

However, the heterogeneity in lower bounds could also point to other factors beyond actual differences in LLM adoption. First, it could highlight nontrivial discrepancies in how authors of different linguistic backgrounds censor suggestions from writing assistants, thereby making the use of LLMs nondetectable for word-based approaches like the one we developed here. It is possible that native and non-native English speakers actually use LLMs equally often, but native speakers may be better at noticing and actively removing unnatural style words from LLM outputs. Our method would not be able to pick up the increased frequency of such more advanced LLM usage. Second, publication timelines in computational fields are often shorter than in many biomedical or clinical areas, meaning that any potential increase in LLM usage can be detected earlier in computational journals. Third, the same is true for journals and publishers with faster turnaround times than thoroughly reviewed, high-prestige journals. Our method can easily be used to reevaluate these results after a couple of publication cycles in all fields and journals. We expect the lower bounds documented here to increase with these longer observation windows.

Given these potential explanations for the heterogeneity in the lower bound of LLM use for scientific editing, our results indicate widespread usage in most PubMed-indexed fields, countries, and journals, including the most prestigious ones. We argue that the true LLM usage in biomedical publishing may be closer to the highest lower bounds we observed, as those may be corpora where LLM usage is the most naïve and the easiest to detect. These estimates are above 30%, which is in line with recent surveys on researchers’ use of LLMs for manuscript writing (*6*). Our results show how those self-reported behaviors translate into real-world LLM usage in final publications.

Last, while our approach can detect unexpected lexical changes, it cannot separate different causes behind those changes, like multiple emerging topics or multiple emerging writing style changes. For example, our approach cannot distinguish word frequency increase due to direct LLM usage from word frequency increase due to people adopting LLM-preferred words and borrowing them for their own writing. For spoken language, there is emerging evidence for such influence of LLMs on human language usage (*32*). However, we hypothesize that this effect is much smaller and much slower. Similarly, we cannot distinguish the influence of different LLMs.

### Related work

Our results go beyond other studies on detecting LLM fingerprints in academic writing. Gray (*25*) described a twofold increase in frequency for the words intricate and meticulously in 2023, while Liang *et al.* (*21*) identified pivotal, intricate, showcasing, and realm as the top LLM-preferred words based on a corpus of LLM-generated text. In contrast, our study performed a systematic search for LLM marker words based on excess usage in published scientific texts. We found 379 style words with highly elevated frequencies in 2024, and all the above examples appear in our list.

Some studies have reported differences in estimated LLM usage between English- and non-English–speaking countries (*18*–*20*), academic fields (*17*), and publishing venues. For example, Liang *et al.* (*21*) estimated the fraction of LLM-assisted papers in early 2024 to vary between 7% for Nature Portfolio papers and 17% for computer science preprints. Our analysis is based on 5 to 200 times more papers per year than these prior works, which allowed us to study LLM adoption with greater statistical power and across a much larger diversity of countries, fields, and journals.

Our approach has two conceptual advantages over previous work: First, all prior studies relied on ground-truth LLM-generated and human-written scientific texts. Such datasets can easily be biased (*16*), with no guarantee that the corpus of LLM-generated texts is representative of all LLM use cases occurring in actual scholarly practice. In contrast, our analysis avoids this limitation by detecting emerging LLM fingerprints directly from published abstracts. Second, our approach is not restricted to LLM usage and can be applied to abstracts from previous years. This allowed us to put the LLM-induced changes in scientific writing into a historic context (e.g., comparing the influence of LLMs to the influence of the COVID-19 pandemic) and to conclude that these changes are without precedent.

### Implications and policies

What are the implications of this ongoing revolution in scientific writing? Scientists use LLM-assisted writing because LLMs can improve grammar, rhetoric, and overall readability of their texts, help translate to English, and quickly generate summaries (*33*, *34*). However, LLMs are infamous for making up references (*10*), providing inaccurate summaries (*35*, *36*), and making false claims that sound authoritative and convincing (*8*, *11*, *12*, *37*). While researchers may notice and correct factual mistakes in LLM-assisted summaries of their own work, it may be harder to spot errors in LLM-generated literature reviews or discussion sections.

Furthermore, LLMs can mimic biases and other deficiencies from their training data (*38*–*41*) or even outright plagiarize (*42*). This makes LLM outputs less diverse and novel than human-written text (*43*, *44*). Such homogenization can degrade the quality of scientific writing. For instance, all LLM-generated introductions on a certain topic might sound the same and would contain the same set of ideas and references, thereby missing out on innovations (*45*) and exacerbating citation injustice (*46*). Even worse, it is likely that malign actors such as paper mills will use LLMs to produce fake publications (*13*).

Our work shows that LLM usage for scientific writing is on the rise despite these substantial limitations. How should the academic community deal with this development? Some have suggested to use retrieval-augmented LLMs that provide verifiable facts from trusted sources (*4*, *47*, *48*) or let the user provide all relevant facts to the LLM to protect scientific literature from accumulating subtle inaccuracies (*8*). Others think that for certain tasks like peer reviewing, LLMs are ill-suited and should not be used at all (*9*). As a result, publishers and funding agencies have put out various policies, banning LLMs in peer review (*49*, *50*), as coauthors (*51*), or undisclosed resource of any kind (*50*). Data-driven and unbiased analyses like ours can be helpful to monitor whether such policies are ignored or adhered to in practice.

In conclusion, our work showed that the effect of LLM usage on scientific writing is truly unprecedented and outshines even the marked changes in vocabulary induced by the COVID-19 pandemic. This effect will likely become even more pronounced in the future, as one can analyze more publication cycles and LLMs are likely to increase in adoption. At the same time, LLM usage can be well-disguised and hard to detect, so the true extent of their adoption is likely already higher than what we measured. This trend calls for a reassessment of current policies and regulations around the use of LLMs for science. Our analysis can inform the necessary debate around LLM policies by providing a measurement method for LLM usage that is urgently needed (*52*, *53*). Our excess word approach could help to track future LLM usage, including scientific (grant applications and peer review) and nonscientific (news articles, social media, and prose) use cases. We hope that future work will meticulously delve into tracking LLM usage more accurately and assess which policy changes are crucial to tackle the intricate challenges posed by the rise of LLMs in scientific publishing.

## MATERIALS AND METHODS

### Dataset

We used the annual PubMed snapshot released in the beginning of 2025 (https://ftp.ncbi.nlm.nih.gov/pubmed/baseline/), containing files from pubmed25n0001.xml.gz to pubmed25n1274.xml.gz. We parsed the XML files as described in our prior work (*30*), applying the same filtering criteria, to extract the abstract texts and some metadata, keeping only complete English-language abstracts with a length of 250 to 4000 characters. This resulted in 24,814,136 abstracts. We then only analyzed papers with publication years from 2010 to 2024, giving us 15,103,888 abstracts for analysis.

For each paper, we defined the affiliation country as the country of the first affiliation of the first author. We assigned papers to the 39 fields based on the journal names (*30*): for example, all papers from all journals containing the word neuroscience (such as *The Journal of Neuroscience* or *Nature Neuroscience*) were assigned to the “neuroscience” field. We used the first names of the first and the last authors to infer their genders via the gender package (*54*). Our gender inference aims to capture perceived gender based on first name and is only approximate. The inference model has clear shortcomings, including limited US-based training data. Moreover, some first names are inherently gender ambiguous. We were able to infer genders only for 55% of the authors. See the study of González-Márquez *et al.* (*30*) for further details.

### Preprocessing

Many abstracts in PubMed data contain strings, usually either in the beginning or in the end, that are not technically part of the abstract text. This can be, for example, “Communicated by:” followed by the name of the editor; or “Copyright” followed by the name of the publisher; or “How to cite this article:” followed by the citation string. Such strings often appear in abstracts from a particular journal starting from a particular year and, in this case, are picked up by our analysis of excess words.

We spent substantial effort to clean the abstracts from all such contaminating strings, using more than 100 regular expressions to find and eliminate them. Overall, 286,744 abstracts were affected by our cleaning procedure. We have also entirely erased 3514 abstracts of errata, corrigenda, correction, or retraction notices (identified based on titles).

We then computed a binary word occurrence matrix using CountVectorizer (binary = True, min_df = 1e-6) from Scikit-learn (*55*), obtaining a 15,103,888 × 362,441 sparse matrix. We focused the subsequent analysis on 273,112 words consisting of at least four letters and composed only of the 26 letters of English alphabet. Note that different strings (e.g., mask and masks) were treated as two distinct words.

### Statistical analysis

To avoid possible divisions by zero, all frequencies were always computed as p=(a+1)/(b+1) , where *a* is the number of abstracts in a given year containing a given word, and *b* is the total number of abstracts in that year.

When computing excess words in year *Y*, we only looked at words with frequencies above 10^−4^ both in year *Y* and *Y −* 1. To do the linear extrapolation, we took the frequencies $p_{-3}$ in year *Y −* 3 and $p_{-2}$ in year *Y −* 2 and computed the counterfactual projection $q=p_{-2}+2\cdot \text{max}\left\{\;p_{-2}-p_{-3},0\;\right\}$ . This way, *q* was always at least as large as $p_{-2}$ (see Fig. 1), resulting in conservative estimates of *r* = *p*/*q* and δ = *p* − *q*.

### Word annotations and lemmatization

We identified 900 unique excess words (surpassing our thresholds on *r* or δ) from 2013 to 2024. Some of these words showed excess usage in multiple years. We sorted the list alphabetically and annotated them as content and style words while being blinded to the year in which they were selected as excess words. We assigned parts of speech (nouns, adjectives, verbs, etc.) in the same way. In case of doubt, the words were discussed between the authors. When we were not certain whether a word was content or style because of ambiguous usage, we did not label this word as either content or style.

To lemmatize the words for fig. S5, we used WordNetLemmatizer() from the Python Natural Language Toolkit (NLTK) library (*56*) and manually added several lemmas such as chatbots → chatbot and circrnas → circrna. We also converted British spelling to the US spelling using a dictionary from https://github.com/hyperreality/American-British-English-Translator.

### Subgroup analysis

For the analysis presented in Fig. 5, we separately analyzed the following subgroups: 50 affiliation countries with the most papers in our dataset; 100 journals with the most papers in our dataset in 2024; 39 research fields taken from the study of González-Márquez *et al.* (*30*); male and female inferred genders of the first and the last authors.

We also analyzed several groups of journals pooled together: (i) *Nature*, *Science*, and *Cell*; (ii) 31 specialized Nature family journals established in 2018 or earlier (from *Nature Aging* to *Nature Sustainability*); (iii) all Frontiers journals called “Frontiers ...” where “...” stands for any sequence of words; (iv) all journals published by MDPI, selected based on their PubMed names “... (Basel, Switzerland).” Subgroups were assigned Δ values only if they contained at least 300 papers in each year from 2018 to 2023.

### LLM usage

We did not use any LLMs for writing or editing the manuscript.

## Supplementary Materials

### This PDF file includes:

Figs. S1 to S7 References
