# Supplementary material for: Delving into LLM-assisted writing in biomedical publications through excess vocabulary
Source: Sci Adv. 2025 Jul 2;11(27):eadt3813. doi: 10.1126/sciadv.adt3813 (PMC12219543; doi:10.1126/sciadv.adt3813)
Supplement: Supplementary file 1 — Figs. S1 to S7 References [file sciadv.adt3813_sm.pdf]

Science Advances
AAAS

Supplementary Materials for

Delving into LLM-assisted writing in biomedical publications through excess vocabulary

Dmitry Kobak *et al.*

Corresponding author: Dmitry Kobak, dmitry.kobak@uni-tuebingen.de

Sci. Adv. **11**, eadt3813 (2025)
DOI: 10.1126/sciadv.adt3813

This PDF file includes:

Figs. S1 to S7
References

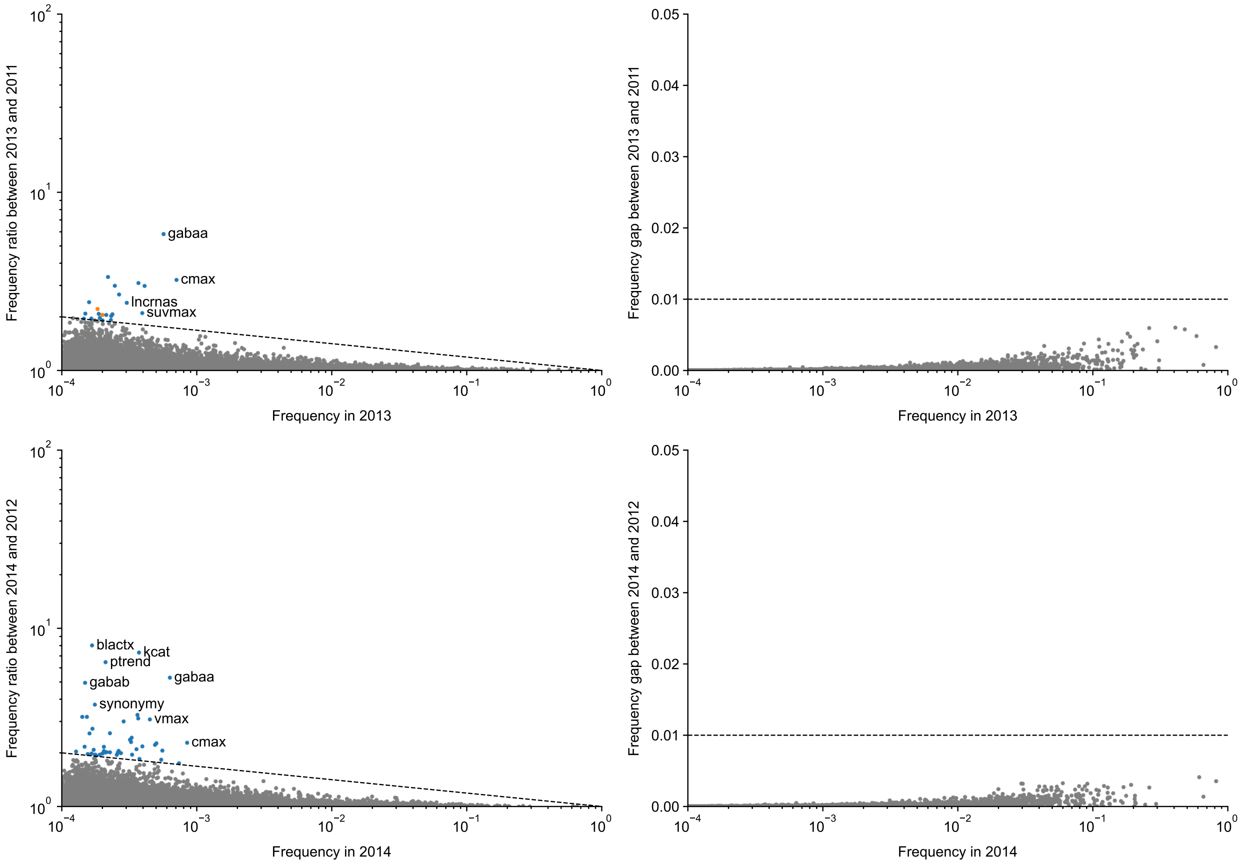

Figure S1: **Excess words in 2013 and 2014.** See Figure 2 for explanations.

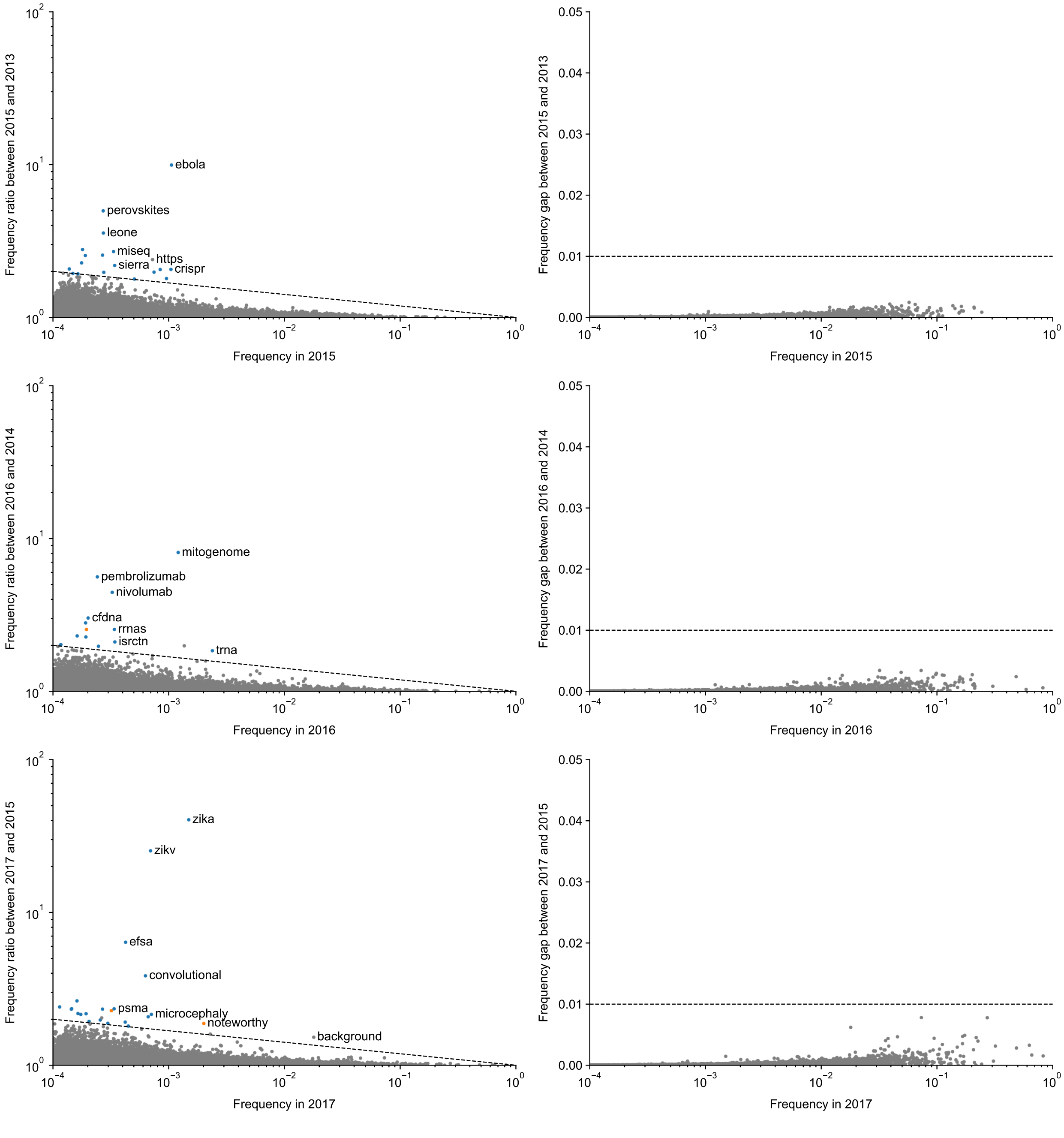

Figure S2: **Excess words in 2015–2017.** See Figure 2 for explanations.

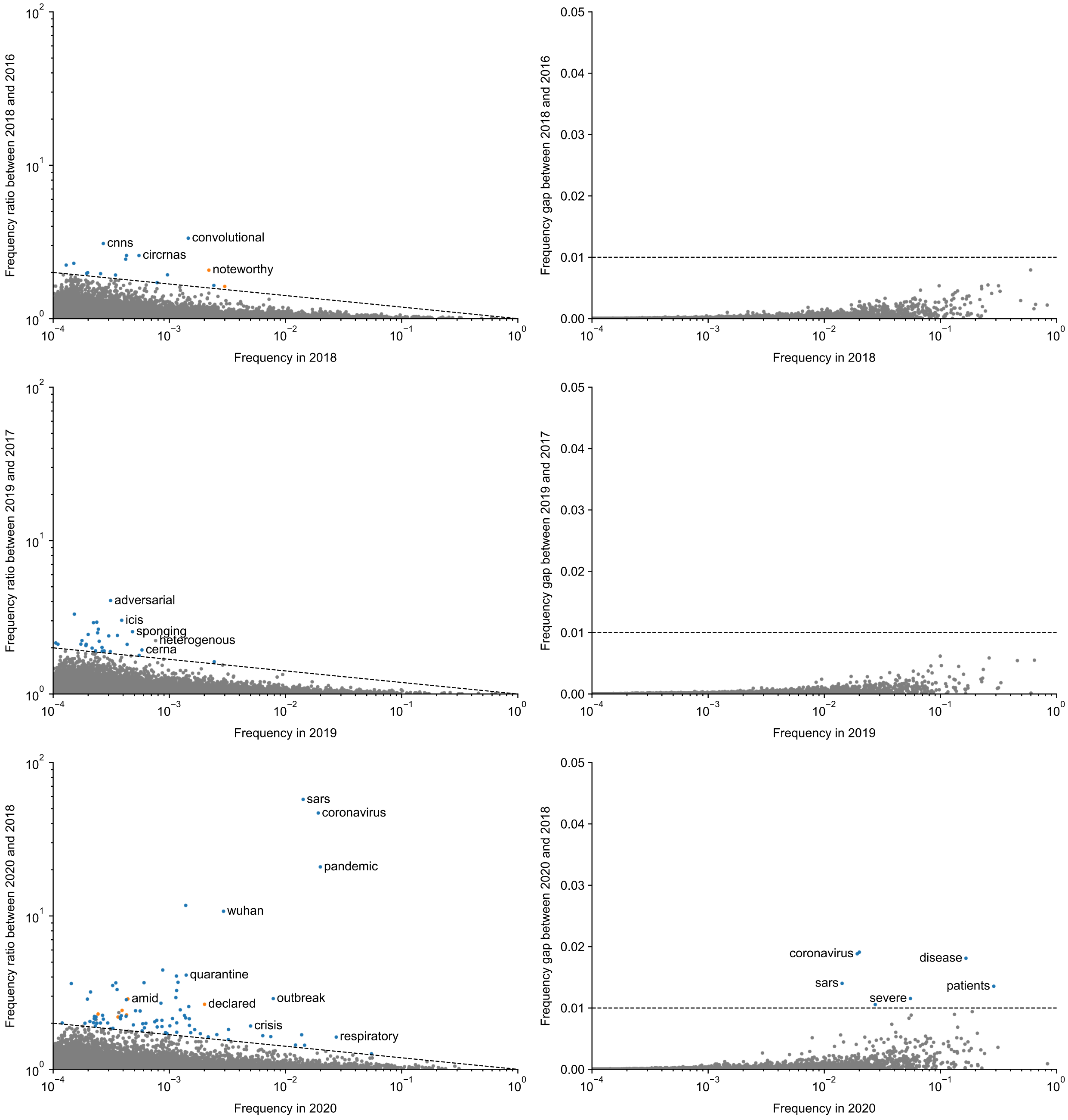

Figure S3: **Excess words in 2018–2020.** See Figure 2 for explanations.

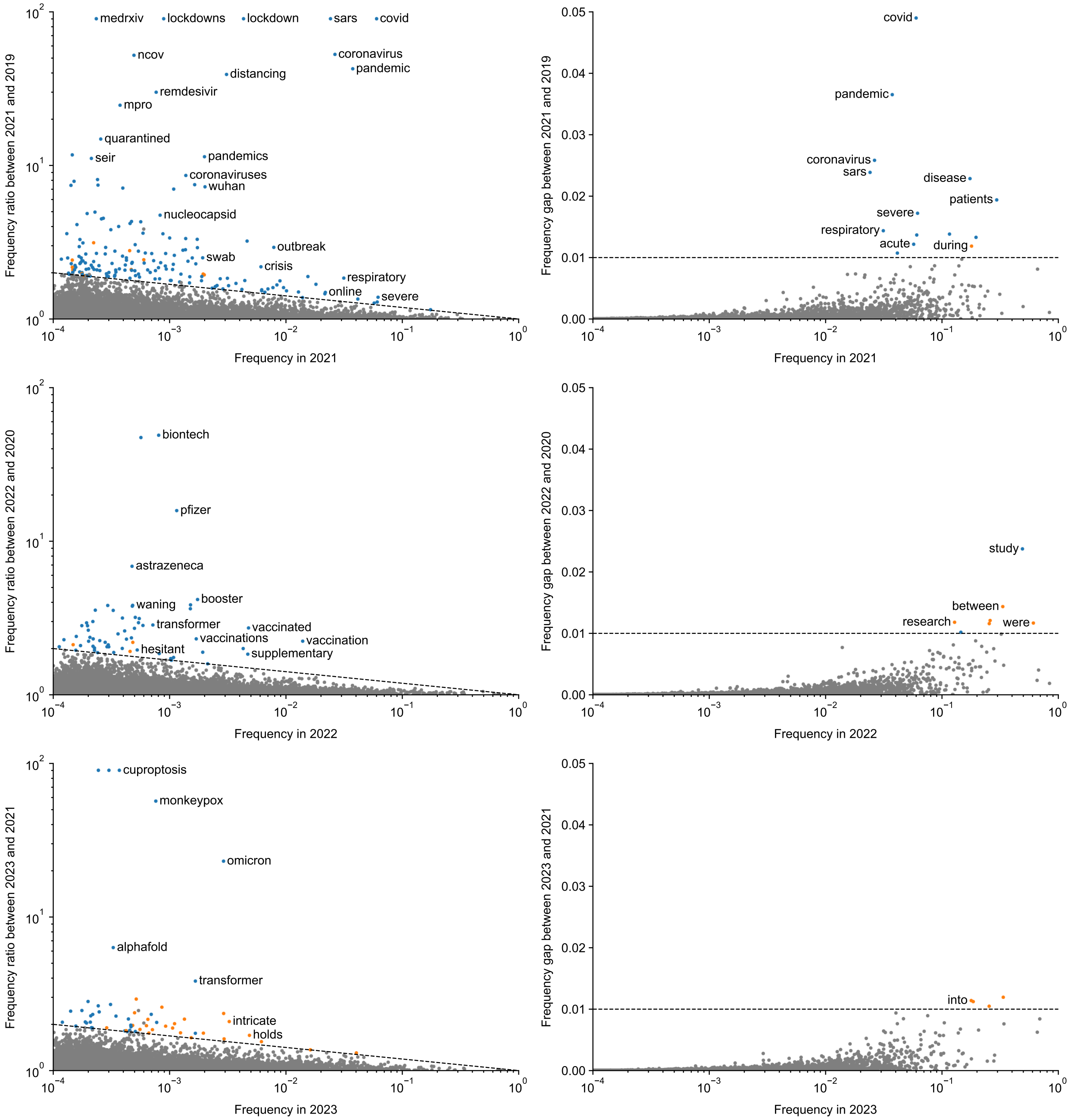

Figure S4: **Excess words in 2021–2023.** See Figure 2 for explanations.

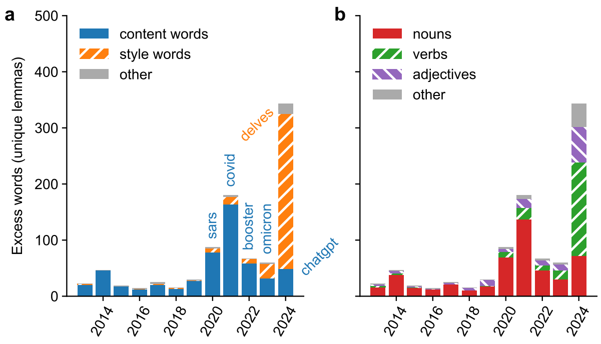

Figure S5: **Number of excess words per year, after lemmatisation.** The same as Figure 3 but showing the number of unique lemmas of excess words, instead of the number of excess words. This counts *delve*, *delves*, *delving*, and *delved* (or *mask* and *masks*) only once.

accentuates, acknowledges, acknowledging, addresses, adept, adhered, adhering, advancement, advancements, advancing, advocates, advocating, affirming, afflicted, aiding, akin, align, aligning, aligns, alongside, amidst, assessments, attains, attributed, augmenting, avenue, avenues, bolster, bolstered, bolstering, broader, burgeoning, capabilities, capitalizing, categorized, categorizes, categorizing, combating, commendable, compelling, complicates, complicating, comprehending, comprising, consequently, consolidates, contributing, conversely, correlating, crafted, crafting, culminating, customizing, delineates, delve, delved, delves, delving, demonstrating, dependability, dependable, detailing, detrimentally, diminishes, diminishing, discern, discerned, discernible, discerning, displaying, disrupts, distinctions, distinctive, elevate, elevates, elevating, elucidate, elucidates, elucidating, embracing, emerges, emphasises, emphasising, emphasize, emphasizes, emphasizing, employing, employs, empowers, emulating, emulation, enabling, encapsulates, encompass, encompassed, encompasses, encompassing, endeavors, endeavours, enduring, enhancements, enhances, ensuring, equipping, escalating, evaluates, evolving, exacerbating, examines, exceeding, excels, exceptional, exceptionally, exerting, exhibiting, exhibits, expedite, expediting, exploration, explores, facilitated, facilitates, facilitating, featuring, formidable, fostering, fosters, foundational, furnish, garnered, garnering, gauged, grappling, groundbreaking, groundwork, harness, harnesses, harnessing, heighten, heightened, hinder, hinges, hinting, hold, holds, illuminates, illuminating, imbalances, impacting, impede, impeding, imperative, impressive, inadequately, incorporates, incorporating, influencing, inherent, initially, innovative, inquiries, integrates, integrating, integration, interconnectedness, interplay, intricacies, intricate, intricately, introduces, invaluable, investigates, involves, juxtaposed, leverages, leveraging, maintaining, merges, methodologies, meticulous, meticulously, multifaceted, necessitate, necessitates, necessitating, necessity, notable, noteworthy, nuanced, nuances, offering, optimizing, orchestrating, outlines, overlook, overlooking, paving, persist, pinpoint, pinpointed, pinpointing, pioneering, pioneers, pivotal, poised, pose, posed, poses, posing, predominantly, preserving, pressing, promise, pronounced, propelling, realm, realms, recognizing, refine, refines, refining, remarkable, renowned, revealing, reveals, revolutionize, revolutionizing, revolves, scrutinize, scrutinized, scrutinizing, seamless, seamlessly, seeks, serves, serving, shaping, shedding, showcased, showcases, showcasing, signifying, solidify, spanned, spanning, spurred, stands, stemming, strategically, streamline, streamlined, streamlines, streamlining, struggle, substantiated, substantiates, surged, surmount, surpass, surpassed, surpasses, surpassing, swift, swiftly, thorough, transformative, typically, ultimately, uncharted, uncovering, underexplored, underscore, underscored, underscores, underscoring, unexplored, unlocking, unparalleled, unraveling, unveil, unveiled, unveiling, unveils, uphold, upholding, urging, utilizes, varying, versatility, warranting, yielding

Figure S6: **Rare excess style words.** All 291 excess style words in 2024 with frequency below 0.02, see Figure 4.

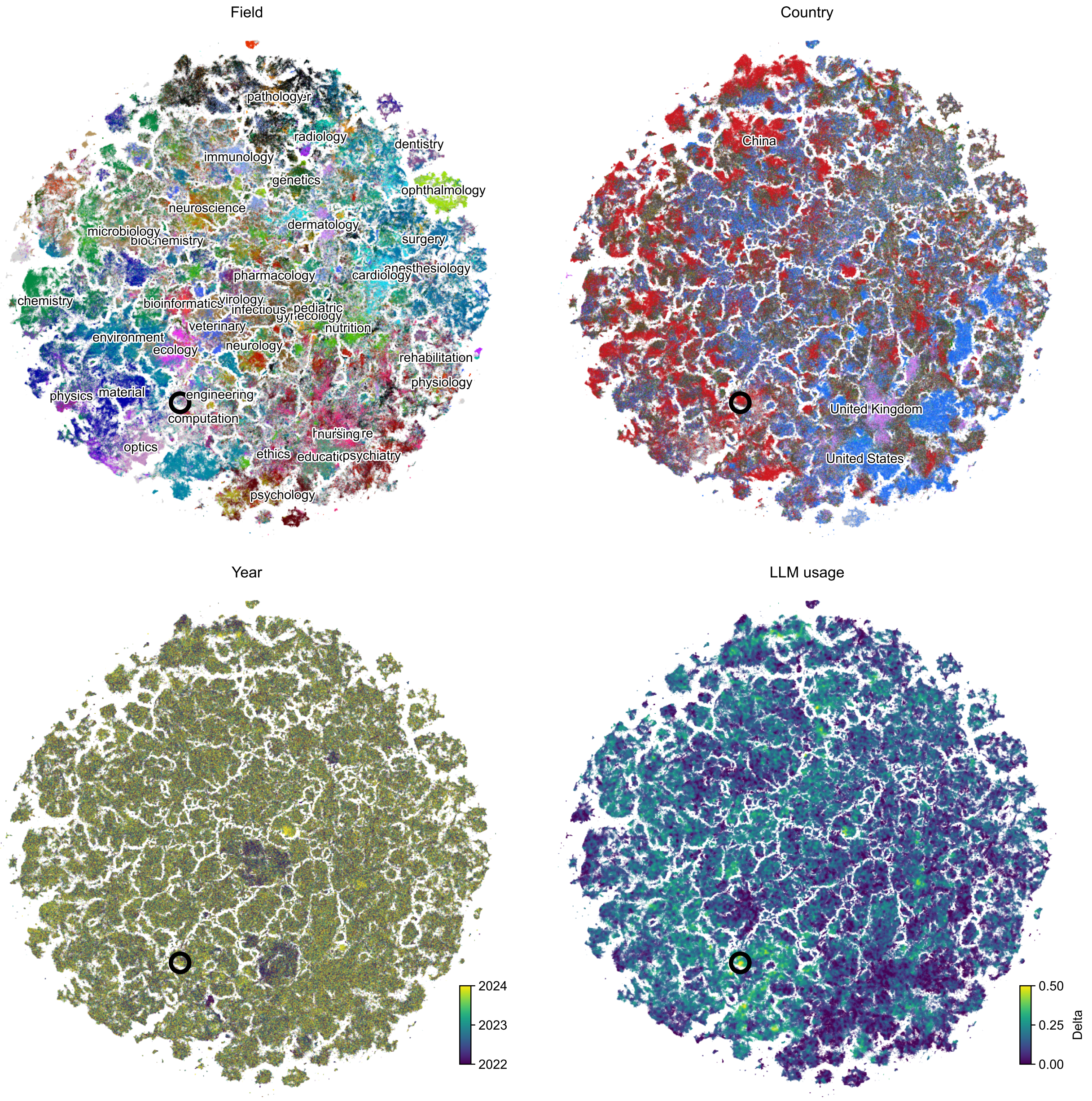

Figure S7: **2D visualisation of 2022–2024 abstracts.** Caption on next page.

Figure S7: (Figure on the previous page.) A 2D visualisation of all 4 109 080 abstracts from 2022 to 2024. Following González-Márquez et al. (*30*), we used the [SEP] token of the pre-trained PubMedBERT model (*57*) to obtain 768-dimensional vector representation of each abstract, and then used *t*-SNE (*31*) to visualize the results in 2D. Top left: colored by field. Labels are positioned at the peak density of each field. Top right: colored by the affiliation country. Bottom left: colored by publication year. Bottom right: colored by local LLM usage. The local LLM usage was defined for each paper based on its nearest neighbors in 2D. Within this local neighborhood, we computed Δ_{rare} and Δ_{common} (see main text for details): For the 100 neighboring 2022 abstracts (and for the 100 neighboring 2024 abstracts), we found the fraction of abstracts containing at least one of the rare (and at least one of the common) excess words. Taking the difference between years yielded Δ_{rare} and Δ_{common}, which we averaged to obtain the final Δ value. This was done separately for each of the embedded abstracts. Note that the regions with high Δ do not consist of only 2024 papers (third panel), but rather of a mix of 2022–2024 papers. This means that our embedding model does not group abstracts based on LLM-associated keywords, but rather by topic. Black circle in all panels highlights one example area with $\Delta \approx 0.50$; it consists of ~400 papers on deep-learning-based object detection, with predominantly Chinese affiliations (83.4%) and with the majority published in *Sensors* (56.3%). Note: a cluster of papers from 2022 (dark blue in the lower-left panel) to the lower-right of the circle contains papers with predominantly Chinese affiliations (79.9%), with many (48.7%) coming from the four journals from the *Hindawi* publisher that were all shut down in 2023 as “heavily compromised by paper mills” (*58*); 28.8% of the papers in this island have been retracted.

REFERENCES AND NOTES

1. V. Bochkarev, V. Solovyev, S. Wichmann, Universals versus historical contingencies in lexical evolution. *J. R. Soc. Interface* **11**, 20140841 (2014).

2. D. Hall, D. Jurafsky, C. D. Manning, "Studying the history of ideas using topic models," in *Proceedings of the 2008 Conference on Empirical Methods in Natural Language Processing*, Honolulu, Hawaii, October 2008 (Association for Computational Linguistics), pp. 363–371.

3. Y. Bizzoni, S. Degaetano-Ortlieb, P. Fankhauser, E. Teich, Linguistic variation and change in 250 years of English scientific writing: A data-driven approach. *Front. Artif. Intell.* **3**, 73 (2020).

4. A. Ahmed, A. Al-Khatib, Y. Boum II, H. Debat, A. Gurmendi Dunkelberg, L. J. Hinchliffe, F. Jarrad, A. Mastroianni, P. Mineault, C. R. Pennington, J. A. Pruszynski, The future of academic publishing. *Nat. Hum. Behav.* **7**, 1021–1026 (2023).

5. V. Berdejo-Espinola, T. Amano, AI tools can improve equity in science. *Science* **379**, 991 (2023).

6. R. Van Noorden, J. M. Perkel, AI and science: What 1,600 researchers think. *Nature* **621**, 672–675 (2023).

7. C. Stokel-Walker, ChatGPT listed as author on research papers: Many scientists disapprove. *Nature* **613**, 620–621 (2023).

8. B. Mittelstadt, S. Wachter, C. Russell, To protect science, we must use LLMs as zero-shot translators. *Nat. Hum. Behav.* **7**, 1830–1832 (2023).

9. G. W. Lindsay, LLMs are not ready for editorial work. *Nat. Hum. Behav.* **7**, 1814–1815 (2023).

10. W. H. Walters, E. I. Wilder, Fabrication and errors in the bibliographic citations generated by ChatGPT. *Sci. Rep.* **13**, 14045 (2023).

11. Z. Ji, N. Lee, R. Frieske, T. Yu, D. Su, Y. Xu, E. Ishii, Y. J. Bang, A. Madotto, P. Fung, Survey of hallucination in natural language generation. *ACM Comput. Surv.* **55**, 1–38 (2023).

12. Y. Zhang, Y. Li, L. Cui, D. Cai, L. Liu, T. Fu, X. Huang, E. Zhao, Y. Zhang, Y. Chen, L. Wang, A. T. Luu, W. Bi, F. Shi, S. Shi, Siren's song in the AI ocean: A survey on hallucination in large language models. arXiv:2309.01219 [cs.CL] (2023).

13. G. Kendall, J. A. Teixeira da Silva, Risks of abuse of large language models, like ChatGPT, in scientific publishing: Authorship, predatory publishing, and paper mills. *Learned Publishing* **37**, 55–62 (2024)M.

14. T. Lazebnik, A. Rosenfeld, Detecting LLM-Assisted writing in scientific communication: Are we there yet? arXiv:2401.16807 [cs.IR] (2024).

15. H. Desaire, A. E. Chua, M. Isom, R. Jarosova, D. Hua, Distinguishing academic science writing from humans or ChatGPT with over 99% accuracy using off-the-shelf machine learning tools. *Cell Rep. Phys. Sci.* **4**, 101426 (2023).

16. R. Tang, Y.-N. Chuang, X. Hu, The science of detecting LLM-generated text. *Commun. ACM* **67**, 50–59 (2024).

17. A. Akram, Quantitative analysis of AI-generated texts in academic research: A study of AI presence in Arxiv submissions using AI detection tool. arXiv:2403.13812 [cs.DL] (2024).

18. H. Cheng, B. Sheng, A. Lee, V. Chaudhary, A. G. Atanasov, N. Liu, Y. Qiu, T. Y. Wong, Y.-C. Tham, Y.-F. Zheng, Have AI-generated texts from LLM infiltrated the realm of scientific writing? A large-scale analysis of preprint platforms. bioRxiv 586710 [Preprint] (2024). https://doi.org/10.1101/2024.03.25.586710.

19. J. Liu, Y. Bu, Towards the relationship between AIGC in manuscript writing and author profiles: Evidence from preprints in LLMs. arXiv:2404.15799 [cs.DL] (2024).

20. P. Picazo-Sanchez, L. Ortiz-Martin, Analysing the impact of ChatGPT in research. *Appl. Intell.* **54**, 4172–4188 (2024).

21. W. Liang, Y. Zhang, Z. Wu, H. Lepp, W. Ji, X. Zhao, H. Cao, S. Liu, S. He, Z. Huang, D. Yang, C. Potts, C. D. Manning, J. Y. Zou, Mapping the increasing use of LLMs in scientific papers. arXiv:2404.01268 [cs.CL] (2024).

22. W. Liang, Z. Izzo, Y. Zhang, H. Lepp, H. Cao, X. Zhao, L. Chen, H. Ye, S. Liu, Z. Huang, D. A. McFarland, J. Y. Zou, "Monitoring AI-modified content at scale: A case study on the impact of ChatGPT on AI conference peer reviews," in *Forty-first International Conference on Machine Learning*, Vienna, Austria, 21 to 27 July 2024.

23. M. Geng, R. Trotta, "Is ChatGPT transforming academics' writing style?" in *Next Generation of AI Safety Workshop at ICML 2024*, Vienna, Austria, 26 July 2024.

24. S. Astarita, S. Kruk, J. Reerink, P. Gómez, Delving into the utilisation of ChatGPT in scientific publications in astronomy. arXiv:2406.17324 [cs.CL] (2024).

25. A. Gray, ChatGPT "contamination": Estimating the prevalence of LLMs in the scholarly literature. arXiv:2403.16887 [cs.DL] (2024).

26. K. Matsui, Delving into PubMed records: Some terms in medical writing have drastically changed after the arrival of ChatGPT. medRxiv 24307373 [Preprint] (2024). https://doi.org/10.1101/2024.05.14.24307373.

27. N. Islam, V. M. Shkolnikov, R. J. Acosta, I. Klimkin, I. Kawachi, R. A. Irizarry, G. Alicandro, K. Khunti, T. Yates, D. A. Jdanov, M. White, S. Lewington, B. Lacey, Excess deaths associated with COVID-19 pandemic in 2020: Age and sex disaggregated time series analysis in 29 high income countries. *BMJ* **373**, n1137 (2021).

28. A. Karlinsky, D. Kobak, Tracking excess mortality across countries during the COVID-19 pandemic with the World Mortality Dataset. *eLife* **10**, e69336 (2021).

29. W. Msemburi, A. Karlinsky, V. Knutson, S. Aleshin-Guendel, S. Chatterji, J. Wakefield, The WHO estimates of excess mortality associated with the COVID-19 pandemic. *Nature* **613**, 130–137 (2023).

30. R. González-Márquez, L. Schmidt, B. M. Schmidt, P. Berens, D. Kobak, The landscape of biomedical research. *Patterns* **5**, 100968 (2024).

31. L. Van der Maaten, G. Hinton, Visualizing data using t-SNE. *J. Mach. Learn. Res.* **9**, 2579–2605 (2008).

32. H. Yakura, E. Lopez-Lopez, L. Brinkmann, I. Serna, P. Gupta, I. Rahwan, Empirical evidence of Large Language Model's influence on human spoken communication. arXiv:2409.01754 [cs.CY] (2024).

33. D. Van Veen, C. Van Uden, L. Blankemeier, J.-B. Delbrouck, A. Aali, C. Bluethgen, A. Pareek, M. Polacin, E. P. Reis, A. Seehofnerová, N. Rohatgi, P. Hosamani, W. Collins, N. Ahuja, C. P. Langlotz, J. Hom, S. Gatidis, J. Pauly, A. S. Chaudhari, Adapted large language models can outperform medical experts in clinical text summarization. *Nat. Med.* **30**, 1134–1142 (2024).

34. T. Zhang, F. Ladhak, E. Durmus, P. Liang, K. McKeown, T. B. Hashimoto, Benchmarking large language models for news summarization. *Trans. Assoc. Comput. Ling.* **12**, 39–57 (2024).

35. L. Tang, I. Shalyminov, A. W.-m. Wong, J. Burnsky, J. W. Vincent, Y. Yang, S. Singh, S. Feng, H. Song, H. Su, L. Sun, Y. Zhang, S. Mansour, K. McKeown, TofuEval: Evaluating hallucinations of LLMs on topic-focused dialogue summarization. arXiv:2402.13249 [cs.CL] (2024).

36. Y. Kim, Y. Chang, M. Karpinska, A. Garimella, V. Manjunatha, K. Lo, T. Goyal, M. Iyyer, FABLES: Evaluating faithfulness and content selection in book-length summarization. arXiv:2404.01261 [cs.CL] (2024).

37. H. Zheng, H. Zhan, ChatGPT in scientific writing: A cautionary tale. *Am. J. Med.* **136**, 725–726.e6 (2023).

38. E. M. Bender, T. Gebru, A. McMillan-Major, S. Shmitchell, "On the dangers of stochastic parrots: Can language models be too big?" in *Proceedings of the 2021 ACM Conference on Fairness, Accountability, and Transparency*, virtual event, 3 to 10 March 2021 (Association for Computing Machinery, 2021), pp. 610–623.

39. R. Navigli, S. Conia, B. Ross, Biases in large language models: Origins, inventory, and discussion. *ACM J. Data Inf. Qual.* **15**, 1–21 (2023).

40. X. Bai, A. Wang, I. Sucholutsky, T. L. Griffiths, Measuring implicit bias in explicitly unbiased large language models. arXiv:2402.04105 [cs.CY] (2024).

41. M. Choudhury, Generative AI has a language problem. *Nat. Hum. Behav.* **7**, 1802–1803 (2023).

42. R. T. McCoy, P. Smolensky, T. Linzen, J. Gao, A. Celikyilmaz, How much do language models copy from their training data? Evaluating linguistic novelty in text generation using RAVEN. *Trans. Assoc. Comput. Ling.* **11**, 652–670 (2023).

43. V. Padmakumar, H. He, Does writing with language models reduce content diversity? arXiv:2309.05196 [cs.CL] (2023).

44. A. J. Alvero, J. Lee, A. Regla-Vargas, R. F. Kizilec, T. Joachims, A. L. Antonio, Large language models, social demography, and hegemony: Comparing authorship in human and synthetic text. *J. Big Data* **11**, 138 (2024).

45. R. Nakadai, Y. Nakawake, S. Shibasaki, AI language tools risk scientific diversity and innovation. *Nat. Hum. Behav.* **7**, 1804–1805 (2023).

46. J. Dworkin, P. Zurn, D. S. Bassett, (In) citing action to realize an equitable future. *Neuron* **106**, 890–894 (2020).

47. P. Lewis, E. Perez, A. Piktus, F. Petroni, V. Karpukhin, N. Goyal, H. Küttler, M. Lewis, W.-t. Yih, T. Rocktäschel, S. Riedel, D. Kiela, Retrieval-augmented generation for knowledge-intensive NLP tasks. *Adv. Neural Inf. Process. Syst.* **33**, 9459–9474 (2020).

48. S. Borgeaud, A. Mensch, J. Hoffmann, T. Cai, E. Rutherford, K. Millican, G. B. Van Den Driessche, J.-B. Lespiau, B. Damoc, A. Clark, D. D. L. Casas, A. Guy, J. Menick, R. Ring, T. Hennigan, S. Huang, L. Maggiore, C. Jones, A. Cassirer, A. Brock, M. Paganini, G. Irving, O. Vinyals, S. Osindero, K. Simonyan, J. Rae, E. Elsen, L. Sifre, "Improving language models by retrieving from trillions of tokens," in *International Conference on Machine Learning* (PMLR, 2022), pp. 2206–2240.

49. J. Kaiser, Funding agencies say no to AI peer review. *Science* **381**, 261 (2023).

50. J. Brainard, As scientists explore AI-written text, journals hammer out policies. *Science* **379**, 740–741 (2023).

51. H. H. Thorp, ChatGPT is fun, but not an author. *Science* **379**, 313 (2023).

52. L. Brinkmann, F. Baumann, J.-F. Bonnefon, M. Derex, T. F. Müller, A.-M. Nussberger, A. Czaplicka, A. Acerbi, T. L. Griffiths, J. Henrich, J. Z. Leibo, R. McElreath, P.-Y. Oudeyer, J. Stray, I. Rahwan, Machine culture. *Nat. Hum. Behav.* **7**, 1855–1868 (2023).

53. R. Heersmink, Use of large language models might affect our cognitive skills. *Nat. Hum. Behav.* **8**, 805–806 (2024).

54. C. Blevins, L. Mullen, Jane, John, ... Leslie? A historical method for algorithmic gender prediction. *DHQ Digit. Humanit. Quart.* **9**, 000223 (2015).

55. F. Pedregosa, G. Varoquaux, A. Gramfort, V. Michel, B. Thirion, O. Grisel, M. Blondel, P. Prettenhofer, R. Weiss, V. Dubourg, J. Vanderplas, P. Alexandre, C. David, M. Brucher, M. Perrot, E. Duchesnay, Scikit-learn: Machine learning in Python. *J. Mach. Learn. Res.* **12**, 2825–2830 (2011).

56. S. Bird, E. Klein, E. Loper, *Natural Language Processing with Python: Analyzing Text with the Natural Language Toolkit* (O'Reilly Media Inc., 2009).

57. Y. Gu, R. Tinn, H. Cheng, M. Lucas, N. Usuyama, X. Liu, T. Naumann, J. Gao, H. Poon, Domain-specific language model pretraining for biomedical natural language processing. *ACM Trans. Comput. Healthc.* **3**, 1–23 (2021).

58. Retraction Watch, Hindawi shuttering four journals overrun by paper mills (2023). https://web.archive.org/web/20230513163806/https://retractionwatch.com/2023/05/02/hindawi-shuttering-four-journals-overrun-by-paper-mills/.
